# Retroperitoneal liposarcoma associated with small plaque parapsoriasis

**DOI:** 10.1186/1477-7819-5-76

**Published:** 2007-07-09

**Authors:** Francesco Tartaglia, Sara Blasi, Monica Sgueglia, Paolo Polichetti, Luciana Tromba, Alberto Berni

**Affiliations:** 1 Department of Surgical Sciences, "La Sapienza" University, viale Regina Elena 324, 00161 Rome, Italy

## Abstract

**Background:**

Extremely rare cases of paraneoplastic syndromes or ectopic production of proteins associated with liposarcoma are reported in literature. Production of Granulocyte-Colony Stimulating Factor, alpha-fetoprotein, paraneoplastic pemphigus and leucocytosis, Acrokeratosis paraneoplastica (Bazex's syndrome) are reported.

The present report describes a case of retroperitoneal liposarcoma associated with small plaque parapsoriasis. Our search in the English literature of such a kind of association did not reveal any case reported.

**Case presentation:**

A 74 year male patient was admitted to our hospital because of the presence of an abdominal mass in right iliac fossa. He also complained of a two-year history of psoriasiform eruptions. The CT scan showed a retroperitoneal pelvic mass. Therefore surgical resection of the tumor was performed. After surgery, the skin eruptions disappeared completely in seven days and so a diagnosis of parapsoriasis syndrome was done.

**Conclusion:**

Parallel disappearing of skin eruptions after surgery, typical clinical picture and not specific histology of the cutaneous lesions suggest the diagnosis of small plaque parapsoriasis. Therefore we propose to add Small Plaque Parapsoriasis to the list of paraneoplastic syndromes associated to liposarcoma.

## Background

Cutaneous paraneoplastic syndromes are a large group of clinical manifestations that can occur in association with an internal malignancy. Characteristically, they follow a course parallel to the tumor and resolve with the successful treatment of primary tumor.

They may precede symptoms associated with the neoplasm and its recurrence will trigger a relapse [1]. The present report describes a case of retroperitoneal liposarcoma associated with small plaque parapsoriasis (SPP). Our search in the English literature of such a kind of association did not reveal any case reported.

## Case presentation

A 74 year old male patient was admitted to our hospital because of subjective sensation of weight in right abdominal lower quadrant lasting 20 days. The patient also complained of a two-year history of skin eruptions.

**Figure 1:**
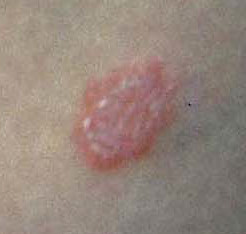
Psoriasiform lesions distributed over trunk and lower limbs (detail). After surgery, skin eruptions disappeared completely in seven days.

A physical examination revealed an abdominal mass in right iliac fossa. It wasn't floating and sore and it had a roundish form. The patient presented anular, erythematous, itchy skin lesions with irregular borders and shaded margins over the trunk and lower limbs. Each lesion was from 1 cm to 5 cm in diameter (Figure 1). No specific abnormal findings were evident on examining his chest and heart. Laboratory findings are detailed in table 1. The white blood cell count was 4600/mm^3^. Hemoglobin was 12,5 g/dl and the platelet count was 123.000/mm^3^. Hematocrit was 35.5%. Serum concentrations of carcinoembryonic antigen (CEA), CA 19-9 and CA 72-4 were within normal limits.

**Table 1:** Summary of peripheral blood samples

WBC	4680/mm^3^	Total protein	6,2 g/dl
Neuthophil	47,2%	Total bilirubin	0,7 g/dl
Eoshinophil	0,86%	GOT	19 IU/l
Basophil	1,28%	GPT	29 IU/l
Lymphocyte	1,88%	LDH	116 IU/l
Monocyte	0,49%	Creatinin	1,00 mg/dl
RBC	4,32 milion/mm^3^	Sodium	140 mmol/l
Hemoglobin	12,5 g/dl	Potassium	4,3 mmol/l
Hematocrit	35,5%		
Platelet	123000/mm^3^		
			
**Tumor markers**	CEA	CA 19-9	CA 72-4
	1,20 ng/dl	8,00 U/ml	1,80 U/ml

Abdominal ultrasonography demonstrated the presence of solid, expansive and roundish mass localized in right iliac fossa with seven centimeters in diameter and a probable origin from cecum. A complete colonoscopy did not reveal endoluminal lesions or compressive signs in any part of large intestine.

**Figure 2:**
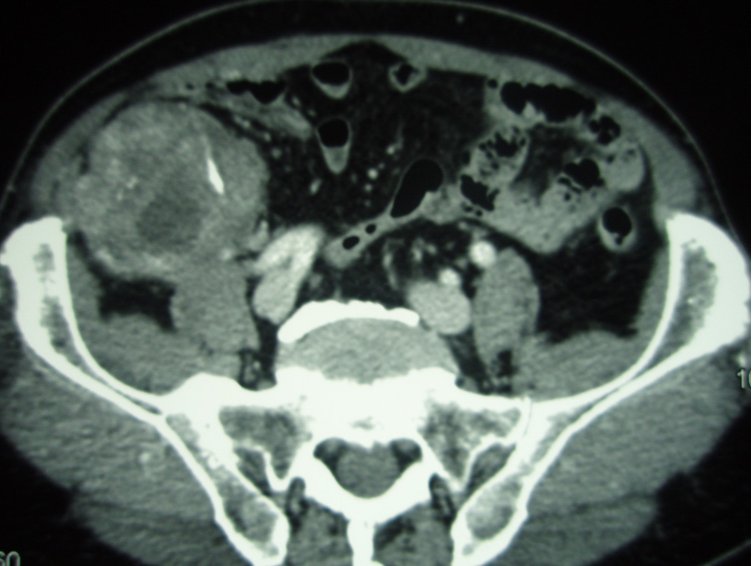
Contrast-enhanced CT scan. The exam showed the extraperitoneal pelvic tumor with a heterogeneous surface, necrotic and calcific areas. The cecum and the right colon are displaced forward and to the left side.

The total body computed tomography showed a solid and extraperitoneal pelvic mass of seven centimeters in diameter with a heterogeneous surface, necrotic and calcific areas and contrast enhancement. It displaced cecum forward and to the left side. It was also revealed a lower and adjacent mass of 2.5 centimeter in diameter with the same characteristics (Figure 2).

Histological examination of a biopsy specimen of a left thigh skin lesion showed hyperkeratosis, orthokeratosis, focal lymphocytic exocytosis and dermic perivascular lymphohistiocytic infiltration.

**Figure 3:**
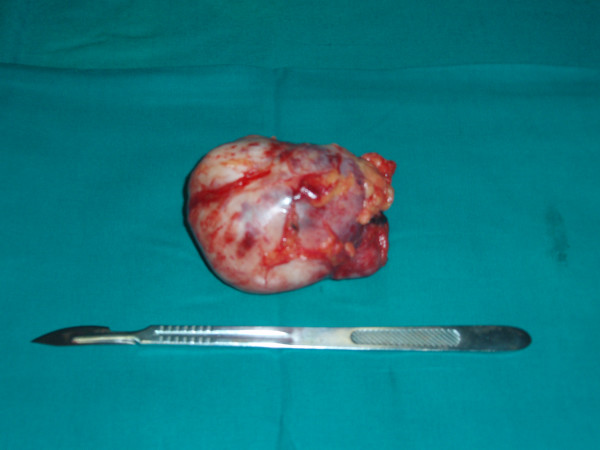
Operative specimen. After surgery the tumor appeared as a whitish roundish mass of 11.5 centimeters in diameter.

**Figure 4:**
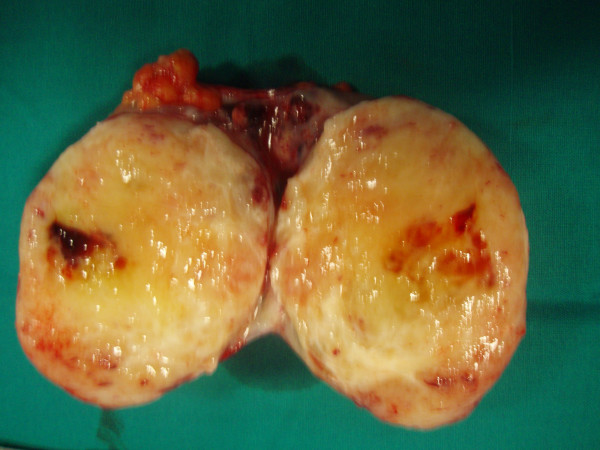
Operative specimen. Tumor section reveals brownish-yellowish areas, haemorrhagic and calcific zones.

Surgical resection of retroperitoneal mass was performed. The second tumor highlighted by CT scan was infact a budding of the larger one, but connected to it. The neoplasm didn't involve iliac vessels, right ureter, psoas or cecum and it showed an own capsule. It was not connected with surrounding organs making quite easy the dissection. Grossly, it appeared as a withish roundish mass of 11.5 centimeters in diameter (Figure 3). The section highlighted brownish-yellowish areas and calcific zones (Figure 4).

Definitive histological diagnosis was consistent with undifferentiated liposarcoma. It was composed predominantly of fusiform cell proliferation with oval-shaped nucleus, finely distributed chromatin and vacuolated cytoplasm. Cell proliferation occasionally showed giant multinucleate elements, an irregular vascular weave and areas of bony metaplasia.

After surgery, skin eruptions disappeared completely in seven days, so a diagnosis of paraneoplastic syndrome was done. Therefore the patient has been subjected to radiation therapy and he is still now undergoing treatment. He doesn't show signs of relapse four months after surgery.

## Discussion

The cutaneous paraneoplastic syndromes reflect the remote effects of cancer. In approximately 1% of cancer patients they are the only sign of unknown malignancy and the clinical manifestations of these dermatoses may precede, coincide with, or follow the diagnosis of cancer. Even if a pathogenic role can be played by tumor derived growth factors, the causes of the skin lesions are often unknown.

The paraneoplastic cutaneous syndromes include reactive erythemas (erythema gyratum repens and necrolytic migratory erythema), vascular dermatoses (Trousseau's syndrome), papulosquamous disorders (acanthosis nigricans, tripe palms, palmar hyperkeratosis, acquired ichthyosis, pityriasis rotunda, Bazex's syndrome, florid cutaneous papillomatosis, the sign of Leser-Trelat, and extramammary Paget's disease), and disorders of hair growth (acquired hypertrichosis lanuginosa) [2].

Therefore extremely rare cases of paraneoplastic syndromes or ectopic production of proteins associated with liposarcoma are reported in literature. Nakamura et all reported for the first time a case of a mesenteric liposarcoma producing G-CSF presenting with iperpirexia and other signs of severe inflammation [3]. Bosco *et al*., described a unique case of relapsing undifferentiated liposarcoma with biochemical, immunoistochemichal and molecular evidence of alpha-fetoprotein (AFP) ectopic production [4]. Sporadic cases of liposarcoma associated with paraneoplastic pemphigus and leucocytosis are also reported [5,6].

Recently liposarcoma has been added to the growing list of malignant neoplasm associated with Acrokeratosis paraneoplastica (Bazex's syndrome) [7]. Our case is of interest because our search in the English literature did not reveal any case reported of retroperitoneal liposarcoma associated with small plaque parapsoriasis. The term parapsoriasis was proposed in the 1902 by Brocq to describe a group of chronic erithematous-squamous skin diseases, all of unknown aetiology [8].

They are divided into three subtypes [9]:

1. *parapsoriasis en gouttes*, today referred as pytirias lichenoides;

2. *parapsoriasis lichenoide*, today referred as parakeratosis variegata;

3. *parapsoriasis en plaque*, today classified in a small plaque parapsoriasis (SPP) and large plaque parapsoriasis (LPP).

Small plaque parapsoriasis affects adult men and is characterized by well bounded oval erithematous lesions, with a slightly desquamative surface, pink-yellowish colour and diameter inferior to 5 centimeters. The skin eruptions, sometimes associated with itchness, are localized over the sides of trunk and limbs, as in our patient. Unlike small plaque parapsoriasis, the psoriasiform lesions in Bazex's syndrome are distributed symmetrically over the acral locations, involving helices of ears, nose, fingers, toes, nails, palms, soles and they tend to spread, if the tumour remains untreated, on the elbows, knees and trunk. Since the typical acral locations were not present in our patient, the diagnosis of paraneoplastic acrokeratosis (Bazex's syndrome) has been excluded.

Histopatologic aspects are not specific and show dermic perivascular lymphocitic infiltration, parakeratosis and sometimes spongiosis [10].

## Conclusion

Parallel disappearing of skin lesions after surgical resection of retroperitoneal liposarcoma supports our diagnosis of paraneoplastic syndrome. Moreover typical clinical picture and non specific histology support the diagnosis of small plaque parapsoriasis. Therefore we propose to add SPP to the list of paraneoplastic syndrome associated with liposarcoma.

## Competing interests

The author(s) declare that they have no competing interests.

## Authors' contributions

FT – main author, was involved in the diagnosis of the case and performed surgery;

SB – concept development and draft the manuscript;

MS – gathered the data and draft the manuscript;

PP – performed literature review;

LT – was involved in diagnosing the case and wrote the manuscript;

AB – supervised the process and finally approved the manuscript for been published.

All authors have read and approved the final manuscript.

## References

[B1] Thomas I, Schwartz RA (2005). Cutaneous paraneoplastic syndromes: uncommon presentation. Clin Dermatol.

[B2] Kurzorck R, Cohen PR (1995). Cutaneous paraneoplastic syndromes in solid tumors. Am J Med.

[B3] Nakamura A, Tanaka S, Takayama H, Sakamoto M, Ishii H, Kusano M, Onizuka Y, Ota S, Mitamura K (1998). A mesenteric liposarcoma with production of granulocyte colony-stimulating factor. Internal Medicine.

[B4] Bosco M, Allia E, Coindre JM, Odasso C, Pagani A, Pacchioni D (2006). alpha-fetoprotein expression in a dedifferentiated liposarcoma. Virchows Arch.

[B5] Krunic AL, Kokai D, Bacetic B, Kesic V, Nikolic MM, Petkovic S, Clark RE (1997). Retroperitoneal round-cell liposarcoma associated with paraneoplastic pemphigus presenting as lichen planus pemphigoides-like eruption. Int J Dermatol.

[B6] Des Guetz G, Mariani P, Freneaux P, Pouillart P (2004). Paraneoplastic syndromes in cancer: Case 2. Leucocytosis associated with liposarcoma recurrence. original presentation of liposarcoma recurrence. J Clin Oncol.

[B7] Gunther Sator P, Breier F, Gschnait F (2006). Acrokeratosis paraneoplastica (Bazex's Syndrome): Association with liposarcoma. J Am Acad Dermatol.

[B8] Brocq L (1902). Les parapsoriasis. Ann Dermatol Syphilgr (Paris).

[B9] Muche JM, Lukowsky A, Heim J, Friedrich M, Audring H, Sterry W (1999). Demonstration of frequent occurrence of clonal T cells in the peripheral blood but not in the skin of patients with small plaque parapsoriasis. Blood.

[B10] Almerio PL, Balengo MG, Calvieri S, Chienti S, Pippione M (2003). Dermatologia evenereologia.

